# Legal and human rights issues in the use of electronic monitoring (using GPS ‘tracking’ technology) in forensic mental health settings in the UK

**DOI:** 10.1177/00258024231174820

**Published:** 2023-05-15

**Authors:** Sepand Malek, Dave Hearn, Thomas Fahy, John Tully, Tim Exworthy

**Affiliations:** 1 6123 University of Nottingham, Nottingham, UK; 2 Psychological Perspectives Salomons Institute, 2238 Canterbury Christ Church University, Canterbury, UK; 3 Department of Forensic and Neurodevelopmental Sciences, Institute of Psychiatry, Psychology and Neuroscience, 4616 King's College London, London, UK; 4 Forensic Psychiatry, Academic Unit of Mental Health and Clinical Neurosciences, School of Medicine, 6123 University of Nottingham, Nottingham, UK; 5 Cygnet Hospital Stevenage, Hertfordshire, UK

**Keywords:** Forensic psychiatry, human rights, law

## Abstract

Electronic monitoring (EM) of individuals has been used by the criminal justice system for the past thirty years, and in the UK, use is on the increase. Its use has been justified as an alternative to prison to reduce recidivism and allowing early release of prisoners, however, the evidence base for this remains mixed. In 2010, it was employed for the first time in a forensic psychiatry setting. A study investigating the effects of EM on leave episodes concluded that EM may improve the speed of patient progress and reduce the length of admission, leading to reduced costs and increased public safety. However, the intervention generated considerable controversy and sparked discussion about ethical concerns. Here, we consider specifically legal and human rights issues that emerge from use of EM in forensic healthcare settings, scrutinising its use in the context of the Mental Health Act and the Human Rights Act. We conclude that EM is legal and justifiable, providing it is used judiciously and with due consideration of concerns for the individual and the given context.

## Introduction

Use of electronic devices to monitor the whereabouts of individuals is referred to in scientific literature as ‘electronic monitoring’ (EM),^1^ although the terms ‘tracking’ and ‘tagging’ are used more widely and interchangeably in the lay literature. Different technologies have been used for EM since its development, with the older radio frequency (RF) technology being largely superseded by Global Positioning System (GPS) technology. GPS technology works through a system of orbiting satellites which constantly send signals to the earth's surface^2^ and allows for real-time monitoring of a wearer's location and movements, in an analogous way to mobile phone technology.

## Electronic monitoring in the criminal justice system

Different forms of electronic monitoring have been used in international criminal justice systems for decades. A large-scale evaluation of use of EM in offenders in the US found that EM reduced the likelihood of failure under community supervision by 31%, relative to offenders placed on other forms of community supervision.^3^ In the UK, use of EM is becoming more widespread with just under 14,000 cases recorded in the year ending March 2021; a 34.3% increase on the previous year.^4^ Its use continues to generate controversy, with critics increasingly questioning its cost-efficiency as well as its ethical basis.^5^ Principles for electronic monitoring in the UK have been published^6^ and these include having a clear purpose for its use and being “used in ways which are tailored and proportionate, flexible and responsive, credible, integrated and transparent and with reference to equality.”^7^ The typical rationale for using EM in the UK is as an alternative to prison and/or to reduce recidivism. In the year to March 2022, 39% of individual uses of EM were as part of bail conditions. In 36% of cases, it formed part of the court sentence (enforcing curfews as part of a community sentence or a suspended sentence, while in 18% of cases, EM was part of post-release conditions allowing early release of prisoners.^4^ A more recent development is remote alcohol monitoring (RAM) as part of Alcohol Abstinence Monitoring Requirements (AAMR).^8^

The evidence for impact of EM on reducing reoffending is mixed. A 2005 review of recidivism studies with a comparison group concluded that, for moderate-high-risk offenders, the data did not support a claim for EM to reduce offending.^9^ A more recent systematic review of studies found that overall EM of offenders was “not associated with statistically significant reduction in reoffending rates”.^10^ However, the report concluded that “EM has been shown to produce positive effects for certain offenders (such as sex offenders), at certain points in the criminal justice process (post-trial instead of prison), and perhaps in combination with other conditions attached (such as geographic restrictions) and therapeutic components”.^10^ Since that report, there have been further reports mentioning the “under-use” of EM devices and a push from The Probation Inspectorate to increase the number of EM devices in use.^11^ Others have commented on the current lack of evidence indicating EM has any “suppression effect” on recidivism, that outlives the period of monitoring.^7^

## Use of EM in a UK forensic mental health setting

In the UK, forensic psychiatry services treat individuals with mental disorder who have committed violent offences or are thought to be at high risk of doing so. Medium secure units (MSUs) occupy a central position in the secure hospital unit hierarchy for patients progressing from high secure care or for many direct admissions from prisons. In 2010, one MSU in London reviewed security arrangements following a series of high-profile absconding incidents, one of which had a tragic outcome.^12^ Subsequently, a ‘tracking’ device for patients on leaves of absence from the hospital was trialled as part of a comprehensive protocol for risk management and recovery.

The procedures involved in the introduction of EM are described in detail elsewhere.^1^ In summary, each patient using the system was allocated their own secure ankle device to be worn when on leave of absence from the hospital. The device worked anywhere a GPS signal could be received and transmitted its location periodically to monitoring software via a mobile phone network. Geographical parameters, known as ‘geo-fences’, could be set enabling the creation of exclusion and inclusion zones (a common sanction for forensic patients). No patient was obliged to wear the device without consent, with the exception of high-risk patients requiring emergency hospital or court transfer. Clinical decisions about the appropriateness of EM in individual patients were made following a specific risk assessment protocol. Informed consent was gained through discussion with the patient, information giving (through an information leaflet), an opportunity to discuss pros and cons (with peers, family and advocates) and finally written consent. It was envisioned that the device would be used primarily in the early stages of leave, when risk of leave violation is the highest.

In a subsequent study investigating the effects of EM on leave episodes and leave violation,^1^ total episodes of leave increased by almost 60% across two follow-up periods after the introduction of EM during leave episodes. Episodes of leave violation reduced in each of the two follow-up periods, significantly so in the second period, after introduction of EM. The use of EM was associated with an increased likelihood of leave being unescorted by hospital staff. These findings suggest potential benefits for speed of patient recovery, reduced length of stay, reduced costs and improved public safety. However, the introduction of EM in this service also gave rise to considerable local, public debate questioning whether it was coercive or risked violating human rights. Equalities groups voiced concern about the impact on black service users who are over-represented in secure settings.^13^

The ethical controversies of GPS tracking are complex and some of these have been aired elsewhere.^14^ The service was acutely aware of these important considerations and established a committee to explore them and sought legal advice. Below, we discuss specifically the legal and human rights issues involved in the use of EM in forensic hospital settings. It is hoped that outlining these issues will support services and service user groups considering use of EM for patients on leave from secure units in the UK, for similar populations in other legislatures internationally and for different patient populations (such as intellectual disabilities, child and adolescent and adult acute services).

## Legal considerations

The use of EM in health is not explicitly prescribed in law. It is, therefore, necessary to analyse whether its use is permissible under existing legislation (Mental Health Act 1983).

Firstly, the use of EM in mental health cannot be a punitive measure. Treatment must have a therapeutic purpose or be necessary for the safety of others in order to be legal. It is therefore necessary to establish a permissible use. There are three sections within the Mental Health Act that contemplate situations which, properly applied, will justify the use of EM. These are:

- As a condition of leave from hospital under section 17 MHA. The MHA allows for “such conditions (if any) as [the responsible clinician] considers necessary in the interests of the patient or for the protection of other persons” (section 17(1) MHA).
- As part of custody whilst on section 17 leave. This permits the patient's responsible clinician to attach conditions, “in the interests of the patient or for the protection of other persons”, to leave of absence, for example, to allow the patient to reside at another hospital or care home or to be kept in custody by a member of staff at that residence.
- As a therapeutic measure under section 63 MHA. This applies to “any medical treatment given … for the mental disorder” not subject to the consent to treatment provisions of the MHA. Medical treatment is given a broad interpretation in the MHA but has to have “the purpose … to alleviate, or prevent a worsening of, the disorder or one or more of its symptoms or manifestations” (section 145(4) MHA). A manifestation of the mental disorder could be verbally or physically aggressive behaviour.

Based on these considerations, it is clear that conditions of leave of absence need to consider the safety of other people as well as the interests of the patient. The most obvious use of EM would be to monitor the whereabouts of a patient when on unescorted leave in the community. It is not unusual for forensic patients who have identified risks associated with a particular person or geographical area to be made subject to exclusion zones as a condition of leave. The need to restrict or at least be aware of the movements of a patient on unescorted leave can equally apply to non-forensic patients. It is for the responsible clinician to consider whether the positive control exerted by having a GPS device monitoring the movements of a patient satisfies the test for appropriate treatment of the patient.

It is possible to consider even wider use under the Mental Health Act, although each use would have to comply with fundamental freedoms protected by the Human Rights Act, which incorporates the provisions of the European Convention on Human Rights into UK law (see below) and it might be difficult to establish that such usage was necessary and proportionate. These might include:

- As a condition of a Community Treatment Order (or CTO). In this instance, the condition would have to be “necessary and appropriate” and the most likely purpose would be for the protection of other persons (section 17B (2) MHA).
- As a condition of a conditional discharge from hospital (sections 37/41). Any condition applied must not be so restrictive as to amount to a deprivation of the person's liberty (SSJ v MM [2018] UKSC 60).^15^

It is notable that, under the Mental Health Act, EM could be used at the discretion of the responsible clinician or an approved clinician. Unlike under the Criminal Justice Act 2003, no court order is required for the use of tracking devices in mental health. The circumstances of each case will be relevant to legal considerations, particularly in light of human rights law considered below. It is contended that what might be appropriate and justifiable in an emergency (for example, an urgent transfer for treatment of a physical condition) or the short term might not be appropriate as a longer term measure.

## Human rights considerations

There is nothing to prevent EM from being used voluntarily by a patient with mental capacity to make such a decision. However, where use engages or potentially engages the fundamental human rights of patients, set out in the European Convention on Human Rights (ECHR), it is necessary to consider whether the rights of the patient prohibit enforced use of EM.

The following rights in the ECHR are those most likely to be engaged:

- Article 3 – freedom from torture, inhuman or degrading treatment or punishment,
- Article 5 – the right to liberty,
- Article 8 – the right to a private and family life.

### Article 3

Torture would not be relevant in this context. Inhuman or degrading treatment means as follows:

- Inhuman treatment is treatment causing intense mental or physical suffering
- Degrading treatment is treatment that arouses feelings of fear, anguish, and inferiority capable of humiliating and debasing victims.

The threshold for breaching Article 3 is high, and has been considered at a European level on a number of occasions. In the leading cases, it has been noted that “the position of inferiority and powerlessness which is typical of patients confined in psychiatric hospitals calls for increased vigilance in reviewing whether the Convention has been complied with” – Herczegfalvy v Austria (1993).^16^ In that case, the Court went on to say “While it is for the medical authorities to decide, on the basis of the recognised rules of medical science, on the therapeutic methods to be used, if necessary by force, to preserve the physical and mental health of patients who are entirely incapable of deciding for themselves and for whom they are therefore responsible, such patients nevertheless remain under the protection of Article 3, whose requirements permit of no derogation … The established principles of medicine are admittedly in principle decisive in such cases; as a general rule, a measure which is a therapeutic necessity cannot be regarded as inhuman or degrading. The Court must nevertheless satisfy itself that the medical necessity has been convincingly shown to exist.”

In Keenan v United Kingdom (2001),^17^ the Court noted that ill-treatment must attain a minimum level of severity if it is to fall within the scope of Article 3. The assessment of this minimum is relative. It depends upon all the circumstances of the case, such as the duration of the treatment, its physical and/or mental effects and, in some cases, the sex, age, and state of health of the victim. In Wilkinson v UK [2006],^18^ the European Court of Human Rights considered what might come within permissible treatment: “The Court recalls that ‘medical necessity’, in this context, is not limited to life-saving treatment. It can also cover treatment, such as anti-psychotic medication, imposed as part of a therapeutic regime. In addition, the decision as to what therapeutic methods are necessary is principally one for the national medical authorities: those authorities have a certain margin of appreciation in this respect since it is in the first place for them to evaluate the evidence in a particular case” (at paragraph: The Law B(2)(a)). This hints at the concept of proportionality which, if these cases were to be considered today, would be a central principle to consider.^19^

Unlike medication, tracking devices are not treatment under section 58 of the Mental Health Act and so, do not provide the patient with the same safeguards (regarding consent to treatment provisions, including Second Opinion Appointed Doctors (SOAD), in the Mental Health Act 1983) as those considered in the above cases. If their use is to be defined in terms of treatment, it falls under section 63 MHA. This section gives the approved clinician broad discretion over the treatment in question and does not require endorsement by a SOAD, unlike section 58 MHA. This means there are fewer procedural safeguards for the use of EM than there would be for enforced medication. However, that does not make it impermissible or unlawful and the use of EM can be mitigated by the application of clear policies about its use on an individual basis.

There are also circumstances where EM is not intended to have a therapeutic purpose. Section 17 MHA provides that the granting of leave can be subject to controls over the patient, including controls over movement. Section 17(3) might particularly apply when the reason for the leave is either unplanned, or not part of habilitation/rehabilitation, for example to attend a specified appointment. Section 17(1) grants a specific power to the responsible clinician to make any period of leave subject to conditions that the responsible clinician considers necessary. Unlike the measures of control available to the approved clinician for treatment under section 63, it does not have to be shown that the use of EM during leave has a therapeutic purpose and it would be sufficient for it to be necessary either in the broader interests of the patient or for the protection of other persons. It may be the case that the use of the tracking device is both treatment for the purposes of section 63 and a reasonable condition of leave. In either case, the approved clinician or responsible clinician will have broad discretion as to whether a tracking device should be used and the relevant test will be one of reasonable “necessity”. For this test to be satisfied, it will be incumbent upon the responsible clinician to demonstrate that the patient's care and treatment have been considered in the round, including:

- Is the use necessary (as opposed to simply desirable)?
- What are the alternatives? This has an inverse relationship with ‘necessity’. Clearly, if there are a range of reasonable alternatives, the necessity for the more restrictive courses of action might be diminished. The least restrictive alternative that is appropriate to the needs of the situation would be the course to adopt.
- Does the patient have capacity to consent?
- Are procedural safeguards in place to avoid arbitrary use; for example, are there effective policies in place and have these been applied to the circumstances of the individual case?

Another key question is: what will happen if the patient refuses to wear the tracking device? It follows from the test of “necessity” that – unless there is a purely voluntary aspect to the wearing of the device – failure to comply with a condition to be subject to the use of EM, could lead to leave not being granted, subject to possible alternative courses of action. It may be that the patient will be in no different a position to that in which he would have been if the option of EM did not exist. In other words, the use of EM allows the early granting of leave and does not objectively penalise those refusing its use. However, if the refusal to wear a tracking device is put down to a lack of insight by the patient into risk or the need for appropriate boundaries, then this would be a factor professionals need to take into account when considering the risk management of the individual.

### Article 5

This article provides people with a ‘limited’ right to liberty. Under the ECHR, people of ‘unsound mind’ can be detained in hospital subject to fair process which establishes that minimum safeguards must be applied and states that certain procedures must be in place to make sure that detention is lawful and not arbitrary or unfair. Until recently, this was effectively provided for by compliance with the Mental Health Act and whilst subject to proper detention under the Mental Health Act, there was little scope for saying that treatment or management for the purposes of the Act warranted separate consideration under this heading.

However, an interesting question about liberty, addressed in the European Court of Human Rights (Munjaz v United Kingdom, 2012),^20^ is whether and to what extent a patient whose liberty is already deprived under the Mental Health Act retains a ‘residual’ liberty that cannot be arbitrarily interfered with. This case is related to the policy on seclusion at a high secure UK mental health hospital. The Court recognised that the concept of residual liberty does exist, although it will require a significant derogation from a patient's reasonable treatment and/or management regime to amount to a breach of any residual liberty. It was found that “seclusion, though coercive, was not imposed on the applicant as a punishment… The length of the applicant's seclusion was foremost a matter of clinical judgment. Seclusion could only continue for as long as those responsible for the applicant's care judged it necessary ….” (Munjaz v UK (2012); paras 70–1).^20^ The Court further noted that the manner of implementing the seclusion policy carried the greatest weight, finding that the hospital's approach was to allow secluded patients the most liberal regime that was compatible with their presentation, and that seclusion was flexibly applied.

The use of EM for a detained patient on section 17 leave is not going to adversely affect that person's residual liberty. Equally the use of EM is unlikely to amount to a deprivation of liberty at all, but rather a restriction on liberty of movement. Human rights case law distinguishes between restrictions on, and deprivation of liberty. The former is governed by Article 2 of Protocol No. 4 of the ECHR whereas the latter is developed from Article 5, ECHR. The difference between the two is “merely one of degree or intensity, and not one of nature or substance” (Guzzardi v Italy (1980); at paragraph 93).^21^ In determining whether a deprivation of liberty has occurred, within the meaning of Article 5, ECHR, “the starting point must be his concrete situation and account must be taken of a whole range of criteria such as the type, duration, effects and manner of implementation of the measure in question” (Guzzardi v Italy (1980); at paragraph 92).^21^

Many have equated use of EM in forensic services with the use of electronic ‘tags’ to monitor the whereabouts and curfew compliance of those who have committed crimes or are suspected of being terrorists. Control orders were originally introduced under the Prevention of Terrorism Act 2005 but have since been replaced by “TPIMs” under the Terrorism Prevention and Investigation Measures Act 2011. The rationale for control orders was to limit a person's interaction with others and with the wider world. They were “made on the basis of suspicion about what they may do in the future …. And the restrictions imposed by the order are for the purpose of prevention and not punishment or deterrence” (SSHD v MB(FC) [2007], per Lord Hoffman, paragraph 48).^22^ Additionally, it was recognised by the courts that the cumulative effects of the obligations imposed by control orders could have the eventual effect of depriving an individual of their liberty, contrary to Article 5 ECHR (JJ v SSHD [2006], E v SSHD [2007]).^23,24^ In other words, a deprivation of liberty could result from the sum of various restrictions on liberty of movement, each of which, individually, would not be considered a deprivation of liberty.

The rationale for the use of EM during patients’ leave from hospital is not only preventive of possible offending but also facilitative, in the sense of permitting the use of leave earlier in the patients’ admissions than might have been considered prudent without additional monitoring. In this respect, its use can be considered analogous to a mental health tribunal's recommendation for the introduction of leave “with a view to facilitating … discharge on a future date” (section 72(3)(a) MHA). It is arguable that EM does not restrict a patient's movement any more than having knowledge of the geographical limit of the section 17 leave or of an exclusion zone they are not to enter and this being complied with. In other words, there is no difference between imposing a condition and being able to monitor objectively compliance with any geographic conditions. In this sense, its use can be compared to urine drug screening tests which provide an objective measure of whether the patient's declaration of abstaining from illicit drug use is correct or not.

However, it could be argued that if the consequence of not complying with EM leads to denial of leave, this could result in an increased period of detention. Such an argument would not hold if the use of EM is to facilitate leave rather than restrict it, since the comparator should be the duration of admission if EM was not available. If using EM can be shown to be part of a necessary risk management strategy that can facilitate early leave, then this would be seen as a positive implementation of technology and a reasonable challenge ought not to arise.

### Article 8

If the reasonable use of EM is unlikely to breach Article 3, what about the less rigorous threshold of Article 8? It was held in Bensaid v United Kingdom (2001)^25^ that treatment which does not reach the severity of Article 3 treatment may nonetheless breach the right to respect for private life in Article 8, if there are sufficiently adverse effects on physical and moral integrity. This follows from the judgment in D v United Kingdom (1997)^26^ which stated “Mental health must also be regarded as a crucial part of private life associated with the aspect of moral integrity. Article 8 protects a right to identity and personal development, and the right to establish and develop relationships with other human beings and the outside world.”

Article 8 is a ‘qualified’ right but any interference has to meet the following criteria:

- being necessary and proportionate,
- in accordance with a procedure prescribed by law, and
- in pursuit of one or more legitimate aims, which are set out on the face of the Convention and include the interests of public safety, the prevention of disorder or crime and protection of the rights and freedoms of others.

As outlined above, the Mental Health Act provides the procedure prescribed by law which satisfies the second part of this test. Having settled on the legitimate aim to pursue, the essential question, therefore, is whether use of EM is necessary and proportionate. Proportionality requires that the implications of the patient wearing an electronic tag be assessed against any detriment that he/she may feel. This will include being monitored and will need to be weighed against the possibility of not having leave from the hospital in the absence of being electronically monitored and the implications of that state of affairs.

There is also the question of restriction of movement. It is already well established that patients can be made subject to restraint as part of custody under section 17 (particularly for emergency transfers or specified appointments) and that they may be subject to exclusion zones when granted leave if certain geographical areas or places of activity are associated with known risk. The question that needs to be added to those under Article 3, therefore, is: Is the use of EM proportionate to the aim it is seeking to achieve? If the question has been properly considered, and the answer is in the affirmative, then the use of the electronic tracker is likely to be a justifiable interference with Article 8.

Munjaz v United Kingdom (2012)^20^ is also applicable here, and is significant in recognising that the Article 8 rights of those who are already subject to controls need to be guarded more carefully. The Court said “the importance of the notion of personal autonomy to Article 8 and the need for a practical and effective interpretation of private life demand that, when a person's personal autonomy is already restricted, greater scrutiny be given to measures which remove the little personal autonomy that is left.” Consequently, when considering the use of EM, any interference with the patient's autonomy will need to be justified on the basis of clear decision making which balances the competing effects of using EM on the patient.

## Conclusion

EM is becoming increasingly prevalent and in 2010 was introduced for the first time in forensic psychiatry. Recent outcomes suggest that EM is more likely to be effective as the technology behind devices improves and becomes more user-friendly for both the monitoring authority and the user, and possibly in select patient groups. We contend that while use of EM as part of the management of patients under the Mental Health Act can be emotive, it is justifiable, provided that it is subject to a reasonable decision-making process. Furthermore, use of EM should be based on an individual risk assessment, with informed consent and in order to facilitate access to leave of absence from the hospital with the aim of pursuing the patient's future discharge. Clinicians, and especially responsible clinicians, should have access to clear policies and procedures which guide them to a decision based on balancing the rights and freedoms of the patient against the need to manage risks that the patient presents to themselves or to other people. Failure to do this could leave the decision to use EM open to challenge for being in breach of a patient's human rights, or a breach of the requirements of the legislation that use is demonstrably necessary.
